# Genome-wide linkage study of atopic dermatitis in West Highland White Terriers

**DOI:** 10.1186/1471-2156-12-37

**Published:** 2011-04-21

**Authors:** Cary A Salzmann, Thierry JM Olivry, Dahlia M Nielsen, Judith S Paps, Tonya L Harris, Natasha J Olby

**Affiliations:** 1 College of Veterinary Medicine, North Carolina State University, Raleigh, NC 27606, USA; 2 Department of Genetics, North Carolina State University, Raleigh, NC 27606, USA; 3 Center for Comparative Medicine and Translational Research, North Carolina State University, Raleigh, NC 27606, USA

## Abstract

**Background:**

Canine atopic dermatitis (AD) is a common, heritable, chronic allergic skin condition prevalent in the West Highland White Terrier (WHWT). In canine AD, environmental allergens trigger an inflammatory response causing visible skin lesions and chronic pruritus that can lead to secondary bacterial and yeast infections. The disorder shares many of the clinical and histopathological characteristics of human AD and represents an animal model of this disorder that could be used to further elucidate genetic causes of human AD. Microsatellite markers genotyped in families of WHWTs affected with AD were used to perform a genome-wide linkage study in order to isolate chromosomal regions associated with the disorder.

**Results:**

Blood samples and health questionnaires were collected from 108 WHWTs spanning three families. A linkage simulation using these 108 dogs showed high power to detect a highly penetrant mutation. Ninety WHWTs were genotyped using markers from the Minimal Screening Set 2 (MSS-2). Two hundred and fifty six markers were informative and were used for linkage analysis. Using a LOD score of 2.7 as a significance threshold, no chromosomal regions were identified with significant linkage to AD. LOD scores greater than 1.0 were located in a 56 cM region of chromosome 7.

**Conclusions:**

The study was unable to detect any chromosomal regions significantly linked to canine AD. This could be a result of factors such as environmental modification of phenotype, incorrect assignment of phenotype, a mutation of low penetrance, or incomplete genome coverage. A genome-wide SNP association study in a larger cohort of WHWTs may prove more successful by providing higher density coverage and higher statistical power.

## Background

Canine atopic dermatitis (AD) is a common, heritable, chronic allergic skin condition that can cause lifelong morbidity in dogs [1,2]. AD is the ninth most common disorder diagnosed in veterinary practice [3], and certain dog breeds such as the West Highland White Terrier (WHWT), with a prevalence as high as 18.7% [4], are more susceptible to the condition than others [5]. In canine AD, environmental allergens such as house dust mites [6] trigger an inflammatory response leading to the development of erythematous macules and papular lesions [7]. Clinical signs usually manifest between six months and three years of age and the chronic pruritus associated with AD leads to excessive licking, alopecia, hyperpigmentation, scaling, lichenification and secondary bacterial and yeast infections [8]. Diagnosis is based on a set of general criteria including chronic or chronic-relapsing pruritus and dermatitis, classical appearance and distribution of lesions, and family history or breed predisposition and elimination of other potential causes for dermatitis such as parasite infection, flea allergy dermatitis, or adverse food reactions [9]. There are currently no laboratory tests that specifically diagnose canine AD [2]. But secondary criteria used to confirm the diagnosis of AD include the demonstration of allergen specific IgE or positive intradermal test to environmental allergens, as well as response to appropriate clinical therapy with glucocorticoids [9]. Treatment includes allergen specific immunotherapy, glucocorticoids and calcineurin inhibitors such as cyclosporine and tacrolimus [2].

Canine AD shares many of the clinical and histopathological characteristics of human AD and is a good animal model for the disease [10] that could prove useful in uncovering additional causative genes in humans. The gene for *filaggrin *(*FLG*), located on human chromosome 1 (HSA 1), has had the most consistent association with AD [11] with two common loss of function mutations initially identified [12], and additional mutations identified subsequently [13]. Filaggrin interacts with intermediate filaments in the cytoskeleton and forms the stratum corneum of the epidermis [14]. The stratum corneum acts as a barrier and locks in essential fluids such as water [15]. However, although *FLG *mutations are associated with human AD, they do not fully account for the disease [12,13]. Investigation of the orthologous canine gene for *FLG*, located on canine chromosome 17 (CFA 17), in a cohort of WHWTs failed to show linkage to canine AD [16] raising the possibility that investigation of the cause of AD in dogs may uncover new candidate genes for the human condition. The study reported here aimed to identify an enriched genetic locus linked to AD in our WHWT pedigrees by testing for linkage to genetic markers across the entire genome. The study was unable to detect any chromosomal regions significantly linked to canine AD in WHWTs using a genome-wide family-based linkage approach.

## Methods

### Recruitment and Sample Collection

WHWTs affected with AD were recruited through referral from veterinary dermatologists and breed clubs across the United States. Pedigrees were collected and the dogs organized into families. Health questionnaires and blood samples were collected from dogs in families containing a minimum of three generations. All protocols were performed with approval from North Carolina State University's Institutional Animal Care and Use Committee. The health questionnaires enquired about each dog's age, pedigree, allergies (including specific questions about the diagnosis and treatment of skin lesions), as well as any family history of allergic disease (if applicable). One of the study's investigators (TJMO), a board certified veterinary dermatologist, determined each dog's affected status by reviewing their health questionnaire and any additional collected information. The diagnosis of AD was made according to standard methods [2] with fulfillment of all the following criteria: family history of pruritic skin lesions; juvenile onset of a pruritic erythematous skin eruption with a characteristic distribution; exclusion of resembling pruritic skin diseases such as non-atopic food allergies, scabies, and skin infections according to standard diagnostic and therapeutic methods; documentation of hypersensitivity against house dust mites, mold or pollen allergens by intradermal testing or allergen-specific IgE serology; response of skin lesions to anti-inflammatory drugs with evidence of efficacy for treatment of canine AD (e.g. oral or topical glucocorticoids, oral cyclosporine or topical tacrolimus). Dogs were considered normal if they reached four years of age with no evidence of clinical signs of AD. Dogs that could not definitively be classified as "affected" or "normal" based on collected information were classified as of "undetermined" status.

Blood samples were collected into acid citrate dextrose (ACD) tubes and shipped overnight on ice to the laboratory where DNA was extracted using the QIAamp DNA Blood Mini Kit (Qiagen, Valencia, CA). DNA concentrations were measured using a ND-1000 NanoDrop spectrophotometer (Thermo Scientific, Wilmington, DE) and stored at -80°C before being genotyped.

### Power Study

Prior to genotyping, a power study was performed to determine the ability of our pedigrees to identify a linkage region containing a recessive locus when considering various penetrance models. The assumption of a recessive trait was based on previous work that suggested a dominant trait was unlikely [17]. Two recessive models were considered. The first involved a highly penetrant mutation in which an individual had a 98% chance of exhibiting the disease trait if they were homozygous for the risk allele. There was also a 1% chance of exhibiting the disease due to other causes (other loci or environmental factors). The second model assumed a locus with a smaller effect size; homozygous individuals had a 20% chance of manifesting the trait while other genotypes had a 5% chance of doing so (again reflecting the possibility of other loci or environmental factors). In both cases the frequency of the risk allele at the causal locus was simulated to be 0.2. Once a genetic model was determined, genotypes for the causal locus and three linked markers were generated using the program "markerdrop" from the Morgan (v.2.8.1) software package [18]. The true pedigree structure was used as a template when generating the simulated data. Once the simulated data was generated, it was analyzed using the "lm_bayes" program of the Morgan (v.2.8.1) software package [18]. This program implements a likelihood-based Markov chain Monte Carlo (MCMC) approach designed for linkage analyses of large pedigrees containing inbreeding loops, which the program allows to remain intact [19]. The same analysis technique was used for the real data. As a model-based approach, this analysis tool requires specification of the genetic model, including allele frequencies of the causal locus. For the analysis of the simulated data, as for the real data, the frequency of the risk allele was set to 0.39, chosen to match the frequency determined in other recessive canine diseases [20]. To gauge the sensitivity of the analysis method to incorrect specification of risk allele frequency, we simulated data using a risk allele frequency of 0.2, and performed the analysis assuming a frequency of 0.39 (the risk allele frequency in the analysis of the real data was also assumed to be 0.39, though other values were also tested). The simulation of data and the subsequent analysis step were repeated 100 times, and the resulting LOD scores recorded. Power was estimated as the proportion of runs in which the LOD score achieved or exceeded a 2.7 significance threshold within the simulated linkage region.

### Genotyping

Whole genome linkage analysis was performed using 288 microsatellite primer sets (Invitrogen, Carlsbad, CA, USA) from the Minimal Screening Set 2 (MSS-2) [21]. In order to perform multiplex PCR, primers were labelled with one of four fluorescent tags (FAM, VIC, NED and PET). Reaction conditions for each marker were optimized for ease of fragment resolution and to prevent wasted resources. The DNA fragments were visualized using an ABI 3730xl DNA Analyzer (Applied Biosystems, Carlsbad, CA, USA) and analyzed using the GeneMapper v.3.7 software (Applied Biosystems, Carlsbad, CA, USA). Genotypes not consistent with familial relationships were removed from further analysis.

### Linkage Analysis

Multipoint linkage analysis was performed using the "lm_bayes" program of the Morgan (v.2.8.1) statistical software package [18]. The trait was modelled as a discrete trait with dogs of undetermined or unknown phenotype assigned a value of unknown.

Chromosomal positions of the heterozygous markers were determined using the online canine genetic linkage map at the University of California, Davis Veterinary Genomics Laboratory (http://www.vgl.ucdavis.edu/dogmap/) and homozygous markers were not included in the linkage analysis. The trait frequency was set to default at 0.61 for the common allele and 0.39 for the risk allele and also altered to assess the effect of different allele frequencies. Marker allele frequencies were calculated using the Case Control tests for Samples with Related Individuals (CC-QLS) [22] and Calculation of Kinship and Inbreeding Coefficients Based on Pedigree Information (KinInbcoef) [23] programs from Mary Sara McPeek's website (http://www.stat.uchicago.edu/~mcpeek/software/index.html). The disease trait was set to default at 98% penetrance, but was lowered to 88% and raised to 99% with no significant changes in the results. LOD scores were calculated for each family separately and then summed across families. Appropriate genome-wide LOD score significance threshold using linkage analysis with canine pedigrees was calculated previously by Gordon et al. [24]. Their results indicated that a threshold of 2.7 yielded a global type-I error of 5% and thus LOD scores greater than 2.7 were considered significant in this study. When chromosomal regions with positive LOD scores were identified, haplotypes of microsatellites in these regions were compared in affected and normal dogs for segregation of homozygosity with affected phenotype. The UCSC Genome Browser (http://genome.ucsc.edu/cgi-bin/hgGateway) was also used to search for candidate genes previously associated with AD in these regions.

## Results

### Sample collection and pedigree analysis

Health questionnaires and pedigrees were collected on 180 related WHWTs. Blood samples were obtained from 108 of these dogs (30 affected, 50 normal, and 28 of undetermined status) including 41 males and 67 females. Using the pedigree information from these WHWTs, three family groups were formed with a minimum of three generations between groups. The smallest of the three families contained nine dogs and the largest family contained 121 dogs. The genotyped WHWTs (22 affected, 40 normal, and 28 of undetermined status) ranged in age from 1 to 16 years, and the average age of affected dogs was 6 years (range 1 year to 13 years) and of normal dogs was 9 years (range 4 years to 16 years).

### Power Study

**Figure 1:**
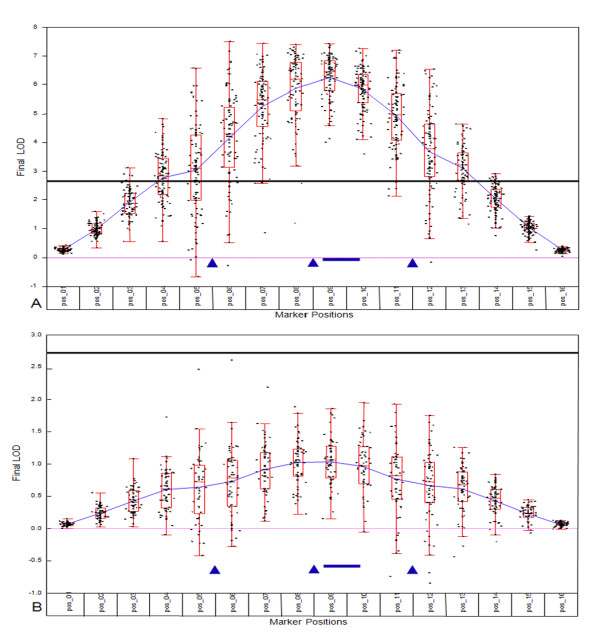
**Power of trait detection**. Simulations to determine the power to detect a highly penetrant mutation (A) and a mutation of low penetrance (B). The bold horizontal line indicates a LOD score of 2.7, bottom bar between the triangles signifies a mutation segregating with AD and the triangles represent locations of microsatellite markers.

In the simulations for which a highly penetrant gene was generated, a LOD score equal to or greater than 2.7 was detected in 100% of the simulation runs, with a maximum score of 7.5 (Figure 1A). For a gene with low penetrance, none (0%) of the simulation runs resulted in a LOD score equal or greater than 2.7, the maximum being 2.6 (Figure 1B).

### Genotyping and Linkage Analysis

Seventy-five WHWTs, spanning the three families were genotyped using the MSS-2 microsatellite makers [21]. Additional dogs were genotyped when preliminary analysis gave positive LOD scores in a region, resulting in 90 of the 108 WHWTs being genotyped on some of the markers. Of the 288 microsatellite markers genotyped, 32 were homozygous and discarded and the remaining 256 were used for linkage analysis. These 256 markers covered the genome with an average inter-marker distance of 8.59 cM, with the largest inter-marker distance being 37.6 cM on CFA 7.

The highest LOD score via linkage analysis was 1.25 on CFA 7, while all other chromosomes had scores less than 0.5. CFA7's peak LOD scores were located between markers FH3972 and FH2973 and spanned approximately 56 cM. Repeated analysis with alteration of both penetrance and risk allele frequency did not result in improved LOD scores.

None of the haplotypes showed segregation between affected and normal dogs in the 56 cM region on CFA 7, but the region did contain the gene for *S100 calcium binding protein A8 *(*S100A8*), one of the units of the calprotectin complex present in epidermal cells [25]. This gene has been associated with human and canine AD previously [26-28].

## Discussion

This report describes a genome-wide linkage study of canine AD in which no chromosomal regions showed significant evidence for linkage. Genome-wide linkage studies using microsatellites have been a useful tool employed to map disease loci in dogs [29-31] however, their power can be limited in complex traits and in diseases where phenotype is difficult to confirm [32]. Our simulations verified that our linkage study had excellent power to detect a highly penetrant mutation. The failure to identify such a locus could suggest that the inheritance of canine AD in WHWTs is of a more complex nature, as is the case in human AD, which has been described as multifactorial and polygenic with a non-Mendelian mode of inheritance [33]. However, linkage analysis has been used in canine epilepsy in which a recent genome-wide linkage scan in Belgian shepherd dogs identified six loci suggesting association with idiopathic epilepsy, a disease of presumed polygenic mode of inheritance with a recessive gene of major influence [34].

The mode of inheritance of canine AD has been investigated in different breeds but a precise mode of inheritance has not been established [16,17,35,36]. We attempted to determine the mode of inheritance using information on complete WHWT litters from normal parents, yet were extremely limited by the difficulty in obtaining detailed phenotypic information on complete litters. Enumeration of affected and normal dogs in seven complete litters did suggest a major recessive genetic influence. Due to the controlled breeding practices used to generate and perpetuate dog breeds, single gene effects can be enriched within one breed or family, allowing their detection with linkage analysis. However, this proved not to be the case with the WHWT pedigrees we worked with.

Genome-wide association studies (GWAS) using single nucleotide polymorphisms (SNPs) are an increasingly popular approach used to identify genetic mutations involved in complex diseases. A GWAS performed in Golden Retrievers with AD identified two SNPs associated with AD located in intronic regions [37]. A further study by the same group to evaluate SNPs in AD candidate genes was performed in Golden Retrievers and seven other dog breeds including the WHWT [38]. A SNP was identified in the *thymic stromal lymphpoietin receptor *(*TSLPR*) gene that was associated with AD in all eight breeds as well as a SNP in *FLG *associated with AD in UK Labradors. Results of this study suggest that different breeds, as well as dogs of the same breed from different geographic regions have different genetic risk factors for AD [38]. As we did not genotype markers on the X chromosome, we cannot exclude the possibility of linkage to a region including *TSLPR *in our WHWT family.

Although a specific chromosomal region linked to AD in WHWTs was not identified, we were able to exclude linkage to the *FLG *locus, corroborating the results of other studies [16,38].

Linkage could not be ruled out in a 56 cM region on CFA 7. The gene *S100A8*, located within this 56 cM region on CFA 7, has been reported to play a role in canine AD [27,28]. Members of the S100 family are pro-inflammatory molecules released by phagocytes [39] and also keratinocytes (epidermal cells) [40] and are present in the human epidermal differentiation complex (EDC) which has been both linked to and shown association with AD in humans [41]. The three microsatellite markers genotyped in the region were separated by 37.6 and 18.7 cM. Genotyping of additional markers in this 56 cM region may definitively rule-out linkage in the region.

Determination of phenotype may also have influenced the results of our study. Affected dogs were diagnosed by one veterinary dermatologist (TJMO) using stringent criteria thereby enhancing the chance of homozygous diagnoses. However, the assignment of normal phenotype is more likely to represent a source of error; it is possible that dogs classified as normal had minimal or seasonal symptoms that were missed by their owners and veterinarians. It is also possible that an apparently normal dog may not have experienced (and possibly never will) the relevant allergenic triggers. While this is a source of concern, normal phenotype was assigned only to dogs older than four years of age and when possible, owners were re-contacted to confirm that there was no change in affection status over the course of the study which spanned several years. Since AD is usually diagnosed before the age of three, this approach limited erroneous identification of normal dogs.

Finally, although the MSS-2 set of microsatellites provides markers across the entire canine genome, homozygosity of some markers in our population of dogs meant that some regions were not covered at high density. A GWAS in a larger cohort of WHWTs may prove more successful by providing higher density coverage of the entire genome and higher statistical power.

## Conclusions

A genetic locus linked to AD in WHWTs was not identified. This could be a result of factors such as environmental modification of phenotype, incorrect assignment of phenotype, a mutation of low penetrance, or incomplete genome coverage.

## Authors' contributions

TJMO and NJO conceived the study and NJO directed the study. JSP contacted owners and breeders, coordinated sample collection, organized and managed pedigrees and paperwork and TJMO assigned phenotypes. TLH and CAS performed laboratory work and linkage analysis. DMN performed the power studies and provided analysis support. CAS and NJO wrote the manuscript with contributions from DMN. All authors read and approved the final manuscript.
